# Different age, different blood parasites - *Acrocephalus* species and their haemosporidian parasites during autumn migration in Central Europe

**DOI:** 10.1016/j.ijppaw.2025.101085

**Published:** 2025-06-01

**Authors:** Nóra Ágh, Szilvia Pásztory-Kovács, Viola Prohászka, Tibor Csörgő, Eszter Szöllősi

**Affiliations:** a HUN-REN-PE Evolutionary Ecology Research Group, Veszprém, Hungary; b University of Pannonia, Behavioural Ecology Research Group, Center for Natural Sciences, Veszprém, Hungary; c University of Veterinary Medicine Budapest, Department of Zoology, Budapest, Hungary; d Hungarian University of Agriculture and Life Sciences, Doctoral School of Landscape Architecture and Landscape Ecology, Budapest, Hungary; e Eötvös Loránd University, Department of Anatomy Cell- and Developmental Biology, Budapest, Hungary; f Eötvös Loránd University, Department of Systematic Zoology and Ecology, Behavioural Ecology Group, Budapest, Hungary; g Hungarian Institute for Forensic Sciences (HIFS), Institute of Forensic Genetics, Department of Reference Sample Analysis, Hungary

**Keywords:** Autumn migration, Haemosporidian infection, *Acrocephalus* species, Age differences, Prevalence

## Abstract

In migratory passerines, the timing of the different phases of the migratory journey is of great importance for the survival prospect of the individuals. Haemosporidian infections could weaken the immune system, decelerate the ability of fat accumulation and potentially influence the migratory behaviour. As a large number of blood parasites is host-generalist there is a potential for cross-species and cross-population transmission with different parasite species during the migratory route of the passerines. In addition, resident birds also interact with different parasite fauna when migratory birds arrive back from their migratory route, therefore, it is important to study what kind of blood parasites migratory birds carry.

We screened three long-distant migratory *Acrocephalus* species during autumn migration in two years. We found that in reed and sedge warblers the overall prevalence of blood parasites was significantly higher in adults than in juveniles, and the prevalence of *Haemoproteus* infections was higher than that of the *Plasmodium* in adults. In contrast, *Plasmodium* infections dominated in juveniles in all the three species. The odds of catching infected juvenile individuals increased during the autumn migration, but infections had no significant effect on the actual body mass of the birds. These results could imply age-related differences in the probability of getting infected with different blood parasite genera. Sampling during migration and exploring the potential differences in parasite species composition and their effects on the migratory behaviour in different age groups can provide valuable insight in answering these questions.

## Highlights

• Prevalence was higher in adult groups, but only weakly biased in different sex groups.
• Distribution of parasite species was age-related.
• Prevalence increased with time during migration in juveniles.
• Infection had no effects on actual body mass.

## Graphical abstract

**Figure:**
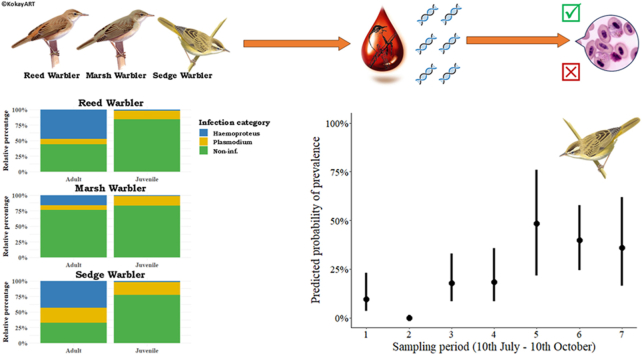

## 1 Introduction

Migration is one of the most studied phenomena in the life of the birds. In recent decades, numerous studies have focused on the effects of migration on the timing of life history events and population dynamics of birds (e.g. Cotton, 2003; Fiedler, 2009; Stępniewska et al., 2024; Thorup et al., 2013; Tøttrup et al., 2012), as well as on changes in phenology and migration strategy due to environmental changes and global warming (Flack et al., 2022; Gienapp et al., 2007; Gordo, 2007; Péron et al., 2007; Visser et al., 2009). Migratory birds have evolved to cope with the physiological and ecological demands of migration (Ishtiaq and Renner, 2020) and have to allocate energy between costly migration and immune defence (Eikenaar et al., 2018; Eikenaar and Hegemann, 2016; Hegemann et al., 2018; Schmaljohann et al., 2022). Because a trade-off between migration and parasitic defence may exist (Altizer et al., 2006; Hegemann et al., 2018) migratory species may be expected to be more parasitized than resident species. In addition, migratory species are more exposed to the parasitic infections during their movements between a range of different habitats (Binning et al., 2017, 2022; Ricklefs et al., 2017). In order to ascertain the potential role of migratory birds in the transfer of parasites and diseases over large distances (de Angeli Dutra et al., 2021; Waldenström et al., 2002), it would be beneficial to investigate whether there are any examples of cross-species transmission observed on the breeding and wintering grounds.

Migratory species especially those that migrate between the temperate and the tropical zones, are the potential sources of cross-species transmission in their breeding and wintering grounds (Garvin and Remsen, 1997; Ishtiaq and Renner, 2020). Though birds migrating along the African-Eurasian flyway carry also their parasites, transmission have not been detected in the temperate zone of Europe in many cases (Hellgren et al., 2009; Kalbskopf et al., 2024). This can be because conditions are not optimal either for the development of blood-sucking arthropods or the parasites in the vectors or in the bird hosts, which prevent the spreading of these parasites in Europe (Lühken et al., 2015; Searle et al., 2013).

Throughout the annual cycle, the possibility of transmission and therefore the prevalence may change within a host population. For example, there were two peaks in *Plasmodium* prevalence in an Oxford population of blue tits (*Cyanistes caeruleus*). The first peak was observed between May and June, while another one between September and October with a strong decline in prevalence in the winter (Cosgrove et al., 2008). Santiago-Alarcon et al. (2013) found that in blackcaps (*Sylvia atricapilla*), the prevalence of haemosporidian parasites decreased from spring to summer and then peaked again later in the same season in the autumn. In another study, prevalence of some *Haemoproteus parabelopolskyi* lineages further decreased during the winter in blackcaps (Pérez-Rodríguez et al., 2015). There are several potential contributing factors behind these patterns. The initially low but constantly increasing prevalence observed during spring in temperate zone is probably caused by the relapses of parasites in chronically infected individuals (Cornet et al., 2014; Valkiūnas, 2005), the increasing number of competent vectors and the transmission of parasites into naïve individuals. This culminates in an autumn peak in prevalence which is mainly result of the fact that also juvenile individuals got infected by that time (Cosgrove et al., 2008). From late autumn parasites retract into the internal organs of the host individuals (Altizer et al., 2006; Deviche et al., 2001; Valkiūnas, 2005). It is crucial to highlight, however, that climate change and the northward expansion of the vector species have the potential to substantially alter the characteristics of this host-parasite-vector system (Garvin and Remsen, 1997; Ishtiaq, 2021; Ortega-Guzmán et al., 2022; Valkiūnas et al., 2022).

Next to the potential changes in transmission and distribution of parasites, another interesting and widely investigated topic is the effects of avian malaria infections on hosts’ physiology, survival and behaviour (Atkinson and van Riper, 1991; Bichet et al., 2020; Jarvi et al., 2001; Mukhin et al., 2016). Surprisingly, the effects of haemosporidian parasites on their hosts during migration have received less attention so far. Among the few studies on the effects of blood parasites during bird migration, Yusupova et al. (2023)found, that infected birds tended to have lower body mass than non-infected individuals but an unexpected positive correlation existed between body mass and the number of lineages infected an individual. Another study on great reed warblers (*Acrocephalus arundinaceus*) tracked with multisensory loggers found that individuals with higher intensity of *Plasmodium* infection migrated for shorter distances than hosts with lower intensity or no infection. Interestingly, these more infected individuals managed to compensate their backlog in distance with shorter resting time (Emmenegger et al., 2021).

In this study we chose three closely related *Acrocephalus* species, the reed (*Acrocephalus scirpaceus*), the marsh (*Acrocephalus palustris*) and the sedge warbler (*Acrocephalus schoenobaenus*), to explore the possible relationship between blood parasites infection, the actual body condition and the arrival time of the individuals. These long-distance migrant species are widespread in Europe and their distribution areas are overlapping. Their migration phenology is well documented in Europe (e.g. Akriotis, 1998; Basciutti et al., 1997; Literák et al., 1994; Nielsen Bo, 2013) and in the Carpathian Basin as well (Kovács et al., 2012; Miholcsa et al., 2009). Despite the increasing number of studies on haemosporidian infections in *Acrocephalus* species, there are several open questions. For example, little is known about the dynamics of infections during breeding and migration in the temperate zone, especially in the case of fledglings and first-year juveniles. Furthermore, most studies examined only a few individuals and often without age separation in the analyses (e.g. Musa et al., 2024; Shurulinkov and Chakarov, 2006; Ventim et al., 2012; Wojczulanis-Jakubas et al., 2012). Therefore, in our two-year study we compared the infection status of adult and first-year individuals and examined the parasite species composition of these three bird species migrating through our study site. Further, we tested if a relationship exists between the actual body mass of the individuals and parasite prevalence within the population during migration. Data was collected directly after the breeding season until the end of autumn migration, so we were able to study the local breeding population and the transmigratory individuals as well.

## 2 Material and methods

### 2.1 Data sampling

We collected data at the Ócsa Bird Ringing Station (Central Hungary: 47°17′ N, 19°12’ E). Our study area is located at the Ócsa Protected Landscape Area (Ramsar Convention, Natura, 2000) in the Danube-Ipoly National Park. The sampling area is a post-glacial bog with mosaic, heterogeneous vegetation ranging from reedbeds to forests, and plays a key role in the life cycle of the *Acrocephalus* species as a breeding and refuelling site during the autumn migration. The ringing station has been collecting data throughout the years using mist nests, following a standard protocol for more than 40 years (Csörgő et al., 2016). Autumn migration monitoring was conducted daily from mid-July to the beginning of November, except in instances of extreme heat, cold, or windy weather conditions. For the molecular work we took blood samples from mid-July until the end of October. Sampling was conducted periodically with alternating 10 days sampling and 10 days break. Blood samples were collected into 96 % alcohol and stored at −20 °C until DNA-extraction.

For species identification we used the description of Svensson (1992) and Demongin (2016). We determined the age of every studied individual: juvenile (hatched in the year of capture) and adult (older) (Svensson, 1992). We measured wing feather length (the flattened maximum wing chord), body mass of the individuals following standardized methods (Csörgő et al., 2016) and estimated the subcutaneous fat deposition by fat scores (the fat scores normally range from 0 to 8, see Kaiser, 1993). We sampled 58 adult (2012: 46; 2013: 12) and 172 juvenile reed warblers (2012: 107; 2013: 65); 72 adult (2012: 59; 2013: 13) and 143 juvenile marsh warblers (2012: 71; 2013: 72) and 80 adult (2012: 53; 2013: 27) and 186 juvenile sedge warblers (2012: 113; 2013: 73) during the study period.

### 2.2 Study species

Reed warblers usually breed in mature *Phragmites* reeds above water or damp ground, including large freshwater reedbeds and stands fringing shallow lakes (Kennerley and Pearson, 2010). After the breeding period they occupy a much wider habitat range and are no longer restricted to reeds. They can even be found away from water sources in secondary bush, acacia, Lantana scrub and forest edge (Kennerley and Pearson, 2010). Their migration period is long-lasting in the Carpathian Basin, the adults depart at the end of July, while juveniles stay here until the middle of October (Kovács et al., 2012). Reed warblers migrate in short steps, flying over the peninsulas and often refuelling their deposits on the stopover sites (Csörgő and Gyurácz, 2009a; Schaub and Jenni, 2001). On the wintering and stopover sites they defend their small, temporary feeding territories (Bibby and Green, 1981; Schaub and Jenni, 2001). This species has two distinct wintering areas: in Southeast and in Western Africa and individuals originating from our region may utilize both areas (Csörgő and Gyurácz, 2009a).

Marsh warblers prefer rank herbage during the breeding season, especially nettles *Urtica*, meadowsweet *Filipendula* and willowherb *Epilobium* and also breed in waterside vegetation and mixed reedbeds with scattered bushes (Kennerley and Pearson, 2010). This species migrates in large steps, and the stopover sites - like our study site - are essential for refuelling their fat deposits (Csörgő et al., 2000). Marsh warblers depart the earliest among the studied species, the adults start their migration in the second half of July, while the latest juveniles leave the site at the end of September (Kovács et al., 2012). This species winters in Southeast Africa, its typical wintering sites are along riverbanks and overgrown streambeds and also below open woodlands and in open forest edges (Kennerley and Pearson, 2010).

Sedge warblers breed mainly in marsh and waterside habitats, nesting in drier edges of reeds and among sedges. This species is less closely linked to lake edges and wetlands than reed warblers and less partial to wetter reedbed areas in standing water (Kennerley and Pearson, 2010). They migrate in wide front to their wintering site in the sub-Saharan region and cross the geographical barriers with a non-stop flight (Bibby and Green, 1981; Trociñska et al., 2001; Zehtindjiev et al., 2003). Their migration starts in July and they stay until September (adults) or mid-October (juveniles) at our study site (Kovács et al., 2012). In Africa their typical wintering quarters are the reeds, sedges, reedmace, *Typha* and occasionally papyrus, around lakes, dams and sewage ponds and along rivers (Kennerley and Pearson, 2010).

The Carpathian Basin is an important passage during the autumn migration of the northern marsh and sedge warbler populations (Fig. 1), thus unlike reed warblers, the individuals trapped at our study site may originate from several different populations (Csörgő and Gyurácz, 2009b). Based on recapture data, the northern populations of reed warblers may avoid the Carpathians on migration (Fig. 1), therefore, we presume that all individuals trapped at the study site are from a homogeneous population breeding within the Carpathian Basin (Csörgő and Gyurácz, 2009a).

**Fig. 1:**
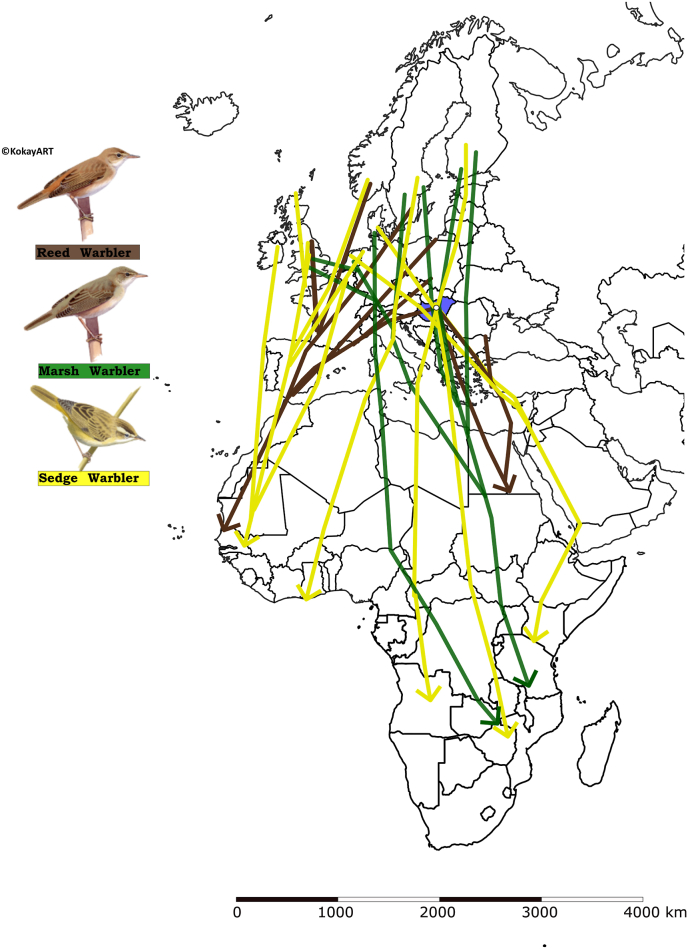
Schematic representation of the migration pathways from the European breeding grounds through the Sahara Desert to the wintering sites in Africa. The three studied *Acrocephalus* species are indicated with different colours (brown lines: reed warblers (*A. scirpaceus*), green lines: marsh warblers (*A. palustris*), yellow lines: sedge warblers (*A. schoenobaenus*); detailed location of wintering site is not shown. The white point in the blue marked country indicates the location of the sampling site. The map has been produced using free vector and raster map data from Natural Earth (naturalearthdata.com) on 1:10 m scale, downloaded from a web platform freely available at https://geojson-maps.ash.ms/. Migration routes were determined using bird ringing data from https://migrationatlas.org.

### 2.3 Molecular methods

DNA was extracted from the blood using two protocols: chloroform-isoamyl alcohol method based on the description of Gemmell and Akiyama (1996) and the Phire™ Animal Tissue Direct Polymerase Chain Reaction (PCR) Kit (Thermo Scientific™). Molecular sexing was performed using the primer pairs P2 – P8 (Griffiths et al., 1998) and the primer pairs CHD1-i9F and CHD1-i9R (Suh et al., 2011). Both of these primer pairs are specific for the *CHD1* (chromodomain helicase DNA– binding protein 1) gene, P2 – P8 amplify amplifies a 390-bp fragment on the W chromosome (in females only) and a 370-bp fragment on the Z chromosome (in both sexes) (Jakubas and Wojczulanis-Jakubas, 2010). With the primers of intron 9 (CHD1-i9F/CHD1-i9R) we detected a 700 bp fragment on the W chromosome and a 1000 bp fragment on Z chromosome (Ágh et al., 2018). These size differences between the fragments are clearly visible separated on 2.5 % agarose gel with electrophoresis.

For the molecular detection of *Haemoproteus* and *Plasmodium* parasites, we used a highly efficient nested PCR method (without modification see: Waldenström et al., 2004). In all PCRs, both negative (ddH_2_O) and positive controls (samples that were previously confirmed to be infected) were included to control for possible contaminations and amplification failures during PCRs, respectively. However, neither negative controls nor positive controls ever showed contamination or amplification failures, respectively. To reduce the risk of false negatives being detected, we screened negative samples twice for blood parasites. To identify the different lineages, all samples with positive amplification were sequenced using the BigDye Terminator v3.1 cycle sequencing kit and sent to a capillary electrophoresis platform (Eurofins Biomi Ltd., Hungary). Sequences were edited and aligned using the program BioEdit (Hall, 1999) and identified to genus and lineage level by comparing sequence data with those of previously identified parasites reported in MalAvi database (Bensch et al., 2009). Parasites with sequences differing by one nucleotide substitution were considered to represent evolutionary independent lineages (Bensch et al., 2004).

### 2.4 Statistical methods

In 2013 we collected the blood samples to other projects, so we did not sample all adult individuals in the pre-planned periods. Therefore, not to bias our statistical results we did not analyse the catching time and biometrical data of adult birds from year 2013 (we calculated only prevalences), but we identified malaria parasites from all infected individuals. During the sequence analysis we found five mixed infections (infected with at least two different haemosporidian lineages) and we could not determine the sequence of nine lineages. These individuals were omitted from the analyses.

First, we calculated the overall prevalence of *Haemoproteus* and *Plasmodium* parasites in all species and in all age and sex categories separately with Sterne methods (Sterne, 1954) using Quantitative Parasitology 3.0 (Reiczigel and Rózsa, 2005) and compared them with Chi-square test. Because of the non-continuous sampling methods (10-day sampling period was followed by another 10-day break) we did not estimate the changes in prevalence in relation to arrival date. In the case of juveniles, we built generalized linear models (GLM) to study the changes of infected/non-infected ratio in relation to sampling periods (as seven level factor), year (as two-level factor) and sex in each host species separately. The models were fitted with binomial error distribution and logit function, where infection status was the response variable and sampling period, year and sex and their interactions were the independent variables. Non-significant interactions were eliminated from the final model. The arrival dates of the individuals in each age group, infected with different parasite morphospecies are shown only graphically (Fig. 3, Fig. 4, Fig. 5), because of the low sample size, we did not analyse them with statistical methods.

To test the possible effects of infection on actual condition of adults and juveniles, we built linear models where body mass was the response variable, and sex group, year (as two-level factor), fat category (as eight level factor), wing length and infection status (no/yes, binomial factor) and their interactions were the independent variables. For this analysis we included body mass data and fat category measured at the first catching, so we had information only about the temporary body condition (hereafter referred as actual body mass and condition). All analyses were performed using R version 4.3.3 (R Development Core TeamR., 2023). For GLM we used “stats” package; for multiple comparisons and p-value corrections, we used “multcomp” package (Hothorn et al., 2008). For visual check of model assumptions (normality of residuals, linear relationship, homogeneity of variance, multicollinearity) we used the “check_model” function in package “performance” (Lüdecke et al., 2021). For the figures we used the “ggplot2” (Wickham et al., 2016) and “ggpubr” packages (Kassambara, 2020).

## 3 Results

### 3.1 Prevalence by age and sex groups

The overall prevalence of avian malaria (*Plasmodium* and *Haemoproteus* species together) in reed warblers was 25.2 % (95 % CI: 19.8–31.4 %; N = 218), in marsh warblers 23.3 % (95 % CI: 17.8–29.7 %; N = 202) and in sedge warblers 33.5 % (95 % CI: 27.6–39.7 %; N = 239) during the whole study period (2012–2013). In 2012, we found significantly higher prevalence in adult reed warblers (Χ^2^ = 32.403, p < 0.001) and sedge warblers (Χ^2^ = 38.314, p < 0.001) than in juveniles. In the case of marsh warblers, the prevalence was nearly equal in the two age groups (Χ^2^ = 0.007, p = 0.933). In juveniles, there was no significant difference in the overall prevalence between males and females (reed w.: Χ^2^ = 0.036, p = 0.850; marsh w.: Χ^2^ = 2.021, p = 0.155; sedge w.: Χ^2^ = 1.392, p = 0.238). In 2012, no sex-related differences were observed in the prevalence of any *Acrocephalus* species in adult age groups (reed w.: Χ^2^ = 0.359, p = 0.549; marsh w.: Χ^2^ = 0.051, p = 0.821; sedge w.: Χ^2^= <0.001, p = 0.999). We did not analyse the adults’ data in 2013, due to the absence of standard sampling methods and an insufficient sample size (see 2.4. Statistical methods for details).

We identified five *Haemoproteus* and six *Plasmodium* morphospecies corresponding to 10 different *Haemoproteus* and 19 *Plasmodium* lineages in our sample (details in Table S1). There were nine lineages which could not to be assigned to any morphospecies and we found one new *Plasmodium* lineages in sedge warbler (P-SW6). The prevalence of the two parasite genera differed markedly in the two age groups of the investigated bird species. In adults, the prevalence of *Haemoproteus* was higher than that of the *Plasmodium* (reed w.: Χ^2^ = 14.750, p = 0.002; marsh w.: Χ^2^ = 66.630, p < 0.001, but see the sedge warbler: Χ^2^ = 4.424, p = 0.219). In juveniles, the pattern was the opposite, the prevalence of *Plasmodium* was higher (reed w.: Χ^2^ = 219.06, p < 0.001; marsh w.: Χ^2^ = 162.770, p < 0.001; sedge w.: Χ^2^ = 189.410, p < 0.001) and we found only a few cases of *Haemoproteus* infections (Table S1).

In juveniles, the prevalence of *Plasmodium* infections was higher in male reed warblers (Χ^2^ = 162.77, p < 0.001; Table S2) and male marsh warblers (Χ^2^ = 63.217, p < 0.001; Table S2), while in sedge warblers the *Plasmodium* prevalence was higher in females (Χ^2^ = 60.418, p < 0.001; Table S2).

### 3.2 Changes in overall prevalence during the migration period of juveniles

We found no significant changes in the prevalence during the autumn migration in juvenile reed warblers and marsh warblers (Table S3, Fig. 2AB). In the case of reed warblers, we had to exclude the data of the first sampling period because of the unsatisfactory fit of the models (Fig. 2A) (i.e. incorrect prediction). In this modified model we found that overall prevalence during the whole study period was significantly higher in 2013 than in 2012 (2012: 11.22 %, 2013: 24.62 %, OR = 3.212, 95 % CI: 1.296–8.216). In the case of sedge warblers, the odds of a caught juvenile being infected increased during the sampling period (from OR_1_ [95 % CI] = 0.152 [0.042–0.422] to OR_5_ [95 % CI] = 8.799 [1.831–4.808], OR_6_ [95 % CI] = 6.237 [1.898–2.479] and OR_7_ [95 % CI] = 5.312 [1.246–2.517]; Table S3, Fig. 2C). The interaction between year and period was not significant in any models, so we eliminated it.

**Fig. 2:**
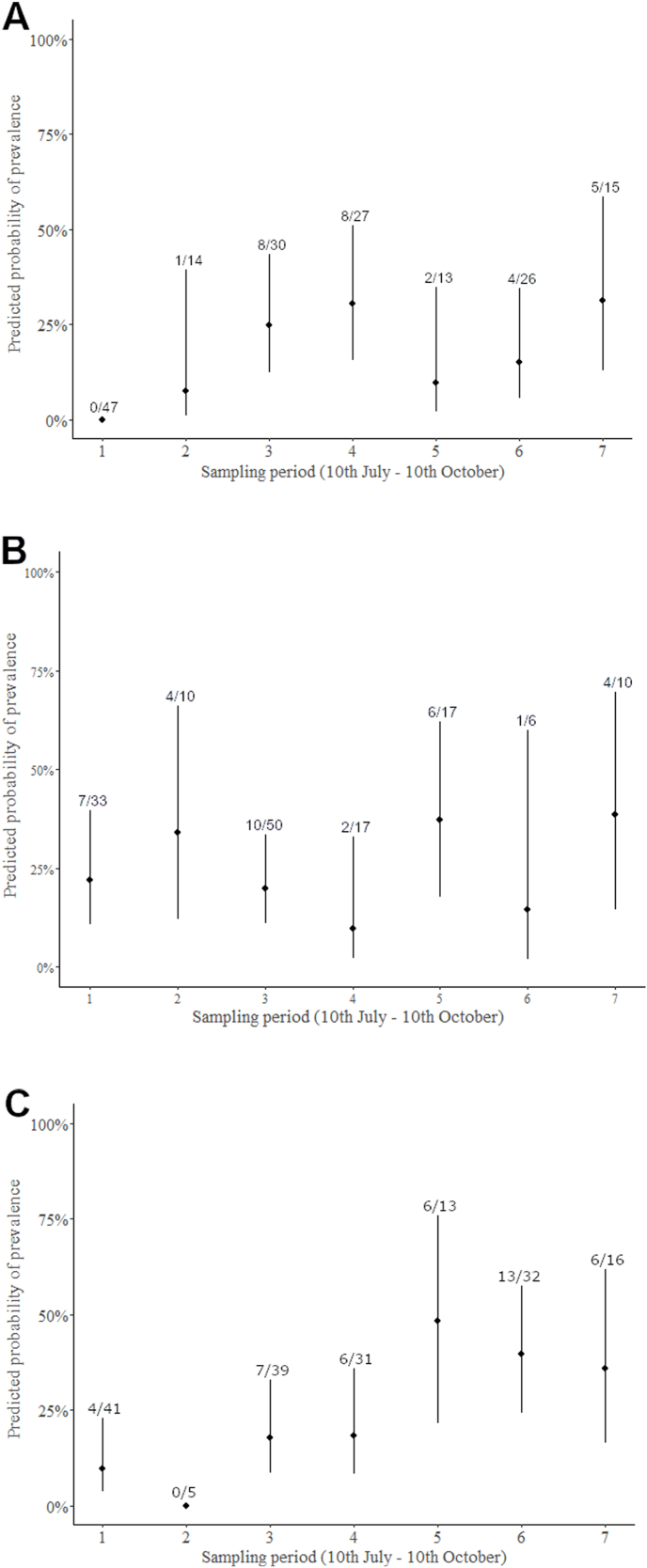
Changes in prevalence during the sampling periods between 10th July and 10th October in the case of Reed- (A), Marsh (B) and Sedge Warbler (C). Sampling was conducted periodically, 10 sampling days were followed by a 10-day-break period. In each species, the duration of the periods was the same. Predicted prevalence means infected individuals/all individuals × 100 in total sample per sampling period in each age groups with 95 % confidence intervals separately, estimated in the fitted model control to the sex and year respectively. Numbers above the bars represent the number of infected individuals per total in a sampling period.

**Fig. 3:**
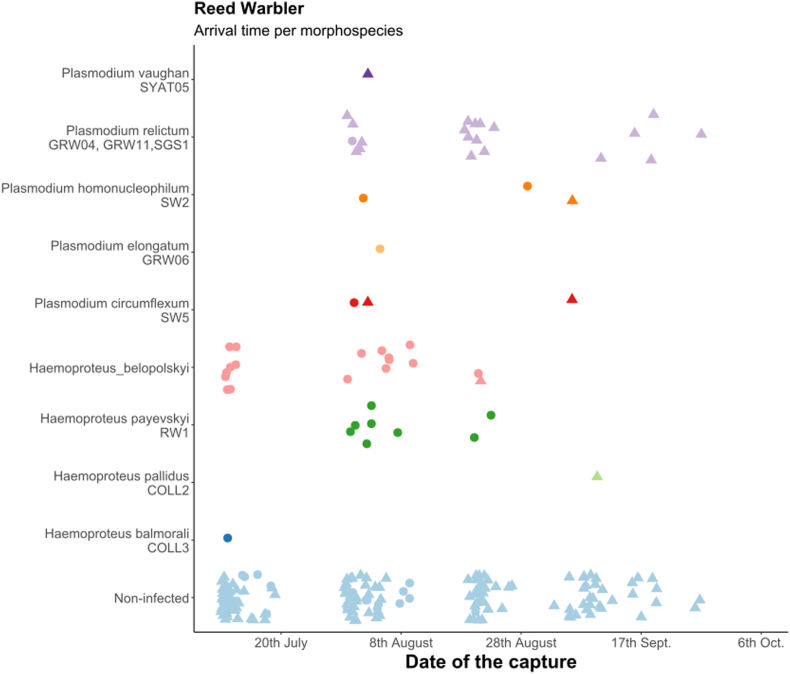
Arrival time of infected reed warblers (Acrocephalus scirpaceus) with different morphospecies from 2012 and 2013 together, age groups shown separately (dots: adults, triangles: juveniles). Light blue dots represent the non-infected individuals.

**Fig. 4:**
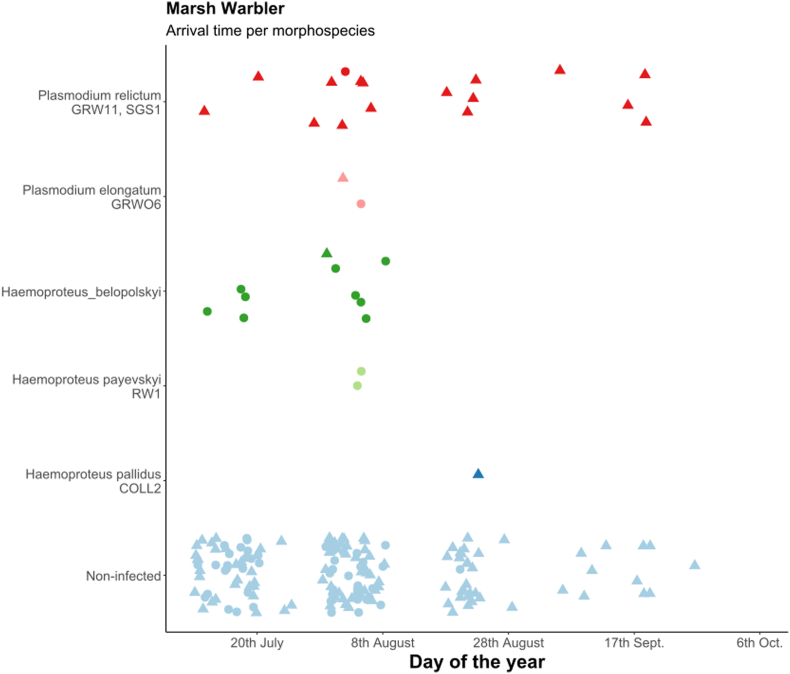
Arrival time of infected marsh warblers (Acrocephalus palustris) with different morphospecies from 2012 and 2013 together, age groups shown separately (dots: adults, triangles: juveniles). Light blue dots represent the non-infected individuals.

**Fig. 5:**
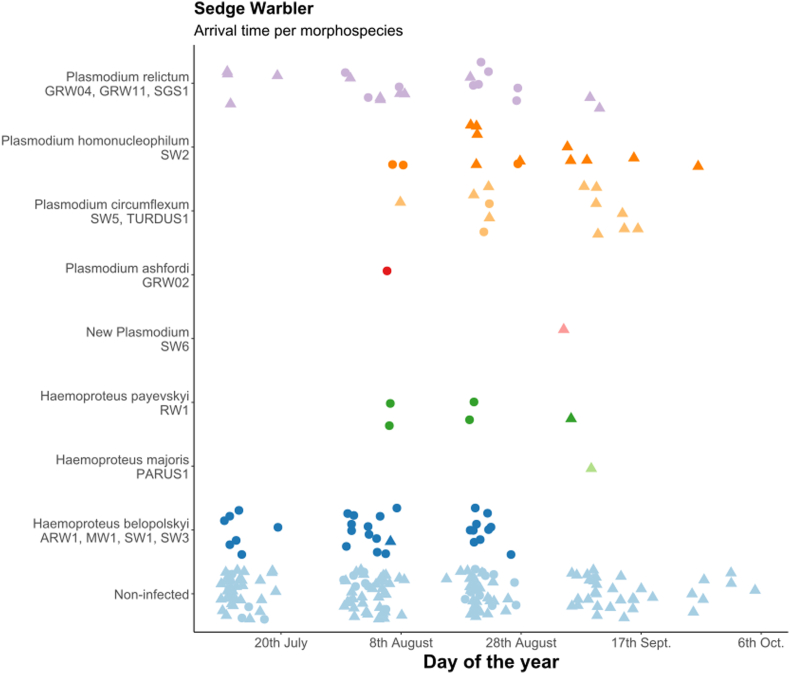
Arrival time of infected sedge warblers (Acrocephalus schoenobaenus) with different morphospecies from 2012 and 2013 together, age groups shown separately (dots: adults, triangles: juveniles). Light blue dots represent the non-infected individuals.

### 3.3 Body mass vs. infection status

We found non-significant difference in body mass between parasitized and non-parasitized individuals after controlling for fat category, wing length, year and sex (reed warbler: −0.186 ± 0.153 g, t-value = −1.220, p = 0.224, marsh warbler: 0.060 ± 0.182 g, t-value = 0.330, p = 0.742; sedge warbler: 0.097 ± 0.121 g, t-value = 0.796, p = 0.427). The interactions between infection status and sex groups were not significant in any case, so we eliminated them from the models.

## 4 Discussion

### 4.1 The importance of studying age-related differences

To better understand the migration phenology and strategies of long-distant migratory bird species, it is important to recognise that there might be great differences in both the timing and the migration strategy of first-time migrating juveniles and experienced adults (Jakubas and Wojczulanis-Jakubas, 2010; Kovács et al., 2011, 2012; Markovets et al., 2008; Stewart et al., 2002). In *Acrocephalus* species, juveniles depart later from the breeding grounds than adults (Jakubas and Wojczulanis-Jakubas, 2010; Kovács et al., 2012; Lehikoinen et al., 2017) and their autumn migration is more prolonged at the European stopover sites. This is because they allocate considerably more time to successfully complete their partial post-fledging moult (Miholcsa et al., 2009; Rguibi-Idrissi et al., 2003) and need more time to accumulate sufficient fat than adults (Stępniewska et al., 2024).

When investigating the potential impacts of blood parasites on migratory strategies and behaviour of the birds (see example Ágh et al., 2022; Emmenegger et al., 2021, 2018; Hahn et al., 2018), it is interesting to examine whether haemosporidian infections have different effects on younger or older individuals during migration. There could be an age-related improvement of immune response against parasites as the immune system matures. Which may manifest in decreasing prevalence/parasitaemia along consecutive seasons and improvement in survival, recovery time, etc. (Bielański et al., 2017; Hammers et al., 2016; Jarvi et al., 2001). However, little is known about the effects of blood parasites on migratory individuals and whether there are differences in their impacts in the different age groups. Haemosporidian parasites were detected in nearly 25–30 % of the samples in this study, showing highest prevalence in sedge warblers (33.5 %) and lower in reed and marsh warblers (25.2 % and 23.3 %, respectively). Our main results showed that prevalence in reed and sedge warblers was significantly higher in adults than in juveniles. Earlier studies also found in reed, marsh and sedge warblers that prevalence was higher in adults than in juveniles during autumn migration but the overall prevalence calculated for the two age groups was different (Fernández et al., 2010; Musa et al., 2024; Waldenström et al., 2002). If the birds survive the acute stage of malaria infection, they could carry the parasites for years or even the whole life, so the higher prevalence and higher diversity in adults might be the result of this accumulation process (Dubiec et al., 2016; Szöllősi et al., 2016; Valkiūnas, 2005).

### 4.2 Age-related differences in species composition

We found that *Haemoproteus* infections were more frequent in adults than in juveniles, while *Plasmodium* infections showed an opposite pattern. This age-related difference is similar to what was observed in some Iberian breeding and transmigrant populations (Ventim et al., 2012) and in North-Germany during the autumn migration (Musa et al., 2024). Unfortunately, in the Iberian study, the arrival or detection date of the birds was not included in the analysis, so it is hard to separate the results between the different seasons. The study site in North-Germany is especially relevant for us, as the local marsh and sedge warbler populations are presumed to arrive at our study site in the second wave of autumn migration for the purpose of refuelling (Csörgő and Gyurácz, 2009b, Csörgő and Gyurácz, 2009c). At these mentioned study sites some of the same *Plasmodium* lineages (P-SGS1, P-GRW11 in juveniles) and *Haemoproteus* lineages (H-RW1, H-RW2, H-SW3 in adults) were detected as we found.

Differences in the prevalence and blood parasite species composition of the two age groups were also found by previous studies conducted on several songbird species. These studies have shown that young and old individuals are differentially exposed to infections (e.g. (Garvin and Remsen Jr, 1997; Granthon and Williams, 2017). First-year old juveniles can be infected already in the nest or during the post-fledging dispersal periods. *Acrocephalus* species build open cup nests, often woven between reed stems or twigs (Kennerley and Pearson, 2010). As a result, chicks are more exposed to the bites of vectors and more frequently parasitized with avian blood parasites than species with closed or cavity nests (Ganser et al., 2020; Lutz et al., 2015). Interestingly, in these two previously mentioned studies higher prevalence of *Haemoproteus* infection was found in open-nest chicks, which is not consistent with our findings in juveniles (i.e. higher prevalence of *Plasmodium* infection was observed in juvenile birds in our study). However, the results of the above and our studies should be compared with caution, given that the data sets of Ganser et al. (2020) and Lutz et al. (2015) were collected in Africa. This suggests that differences in the composition of the vector and parasite fauna, and the microclimatic parameters of the habitats in the European breeding and African wintering sites could potentially answer the differences detected in the infection patterns of the two bird populations.

On the other hand, it is possible that differential habitat use of the two age groups explains this apparent discrepancy in parasite prevalence. In *Acrocephalus* species, the first-year-old birds have an important dispersal movement before migration, during which juvenile males spend the majority of their time with searching for potential future breeding sites (Bulyuk et al., 2000; Mukhin, 2004). It is interesting to note that no previous observations have been made for age-dependent differences in habitat use during the dispersal period or autumn migration. However, Andueza et al. (2014) found that on an autumn stopover site in the Iberian Peninsula, migrating first-year birds exhibited larger home ranges (i.e. small but non-defended areas in which individuals perform their normal activities (Chernetsov and Mukin, 2006)) than either local or migrating adults. Additionally, juveniles showed lower fat deposition rates, while adult birds appeared to gain fat rapidly and then continued their migration faster than juveniles. These differences may cause differential exposure and sensitivity to vectors and parasites.

Interestingly in another study, Shurulinkov and Chakarov (2006) found only *Haemoproteus* species in adult and first year birds and argued that there was no local transmission of *Plasmodium* species at the three breeding sites next to the Black-Sea. However, it is important to note, that the number of sampled individuals was low, and the samples were collected in July only, during the dispersal period but before the autumn migration of the birds. It is worthwhile to note that the majority of their previously identified lineages and parasite species were observed in adult birds (Stanković et al., 2019; Valkiunas et al., 2014). Only transmission of H-RW2 lineages has been documented in juvenile reed warblers (Dimitrov et al., 2018) making it difficult to determine where parasite transmission takes place.

A previous study conducted on reed and sedge warblers at the potential wintering site of these species in Nigeria detected several of the lineages that we also identified from our birds (Waldenström et al., 2002). This suggests that it is possible that *Haemoproteus* lineages detected from adults mostly transmitted in the African wintering areas of *Acropcephalus* species and the reason why we could not detect them from juvenile birds is because no transmission takes place in the European breeding sites.

To support our hypothesis, we checked the transmission areas of the lineages detected in our study (Table S1). We found that the *Plasmodium* lineage P-TCHSEN01 is probably transmitted in Africa because it was detected only from migratory and African resident species (Harvey and Voelker, 2017). On the other hand, three *Haemoproteus* (H-ARW1, H-SW1, H-SW3) and one *Plasmodium* lineages (P-GRW04) can possibly be transmitted in some areas of Europe as they were found in both European resident and juvenile birds before their first migration to Africa (Bensch et al., 2000; Ciloglu et al., 2020; Dimitrov et al., 2010; Hellgren et al., 2007; Šujanová et al., 2021; Waldenström et al., 2002). Though there are some indications that the *Haemoproteus* lineage H-MW1 (Casanueva et al., 2012) is transmitted in some European breeding sites too, this lineage was only found in adult *Acrocephalus* species in our case (Table S1).

The fact that two *Haeomproteus* lineages (H-SW1, H-RW2), and four *Plasmodium* lineages (P-GRW04, P-SW2, P-SW5, P-SGS1) were detected from juvenile birds at our study site support their transmission at the European breeding grounds. European transmission of some of the lineages detected from adult birds was already confirmed by previous studies (H-RW2, P-GRW04, P-SW2 and P-SGS1, see e.g. Ellis et al., 2020; Hellgren et al., 2007; Musa et al., 2024; Ventim et al., 2012), except for the case of *Haemoproteus* lineage H-SW1, which was the most prevalent blood parasites detected in adult sedge warblers in southern Poland (Biedrzycka et al., 2015). We found this lineage in one juvenile reed and one juvenile sedge warblers too, but more focused data sampling is needed to map the transmission areas of H-SW1 more precisely.

The parasite species detected from our birds are predominantly generalists or commonly associated with the *Acrocephalus* genus (see Table S1). However, we detected two *Haemoproteus* lineages (H- COLL2 and H-COLL3) that were not observed in the *Acrocephalus* genus previously. These lineages were found in three of our samples: in two adult and one juvenile individuals. Both of these lineages were already proved to be transmitted in European breeding sites as H-COLL2 was detected also from a juvenile song thrush (*Turdus philomelos*) and H-COLL3 was detected from juvenile pied and collared flycatchers (*Ficedula hypoleuca* and *F. albicollis*) in Sweden (Ellis et al., 2020).

Age-related differences in the effects of *Haemoproteus* and *Plasmodium* lineages on survival of the birds could also explain the different infection patterns of the two age groups. If we assume that the survival rate is lower in the case of *Haemoproteus* infection, this could explain why higher prevalence of *Plasmodium* species was found in the sampled juveniles. However, this scenario is less likely, since based on earlier studies in different bird species, *Plasmodium* infection has been shown to cause higher mortality than *Haemoproteus* spp. (e.g. Atkinson and Samuel, 2010; Atkinson and van Riper, 1991; Palinauskas et al., 2008, 2016).

We found no significant sex-related differences in the overall prevalence, though in juveniles, *Plasmodium* prevalence was slightly higher in male than in female reed and marsh warblers, but the opposite trend was found in sedge warblers (Table S2). We have to note, however, that because of the low sample size, we were not able to compare the prevalence in adults, though prevalence of *Plasmodium* and *Haemoproteus* seems to be rather equal in males and females (Table S2). This pattern is interesting, because based on an extensive dataset, only *Haemoproteus* infections were biased towards females in a meta-analysis (Valdebenito et al., 2024). It is important to note, however, that this study used data exclusively from adult males and females, and the differences found in prevalence showed seasonality between breeding and non-breeding periods.

### 4.3 Changes in prevalence during migration and association with body mass

In our study we compared infected and non-infected juvenile individuals, and found no considerable difference in the actual body mass of *Acrocephalus* warblers. This result may suggest that malaria infection had no relevant disadvantageous effects on the body condition of juvenile *Acrocephalus* species during migration. Fat accumulation and the ability to increase the body mass are key elements of the migration strategy of these species, especially of marsh and sedge warblers, which need to deposit large amount of fat before they leave their European stopover sites. In contrast, reed warblers migrate in small steps and probably had more disruptions during migration (Csörgő and Gyurácz, 2009a; Kennerley and Pearson, 2010). When we compared the actual body mass of infected and non-infected individuals, we most likely saw only the consequences of the infections, as haemosporidian infections are shown to cause delay in fat accumulation and body mass gain (Krams et al., 2013; Marzal et al., 2013; Palinauskas et al., 2008), though the effects of infections should be stronger during the acute phase.

In our study we were only able to analyse if fat deposit or the actual body condition of the birds are linked to the prevalence of blood parasites. To get a more accurate overview of this complex system, we need detailed investigations, for example additional physiological data from the host species from the beginning of the infection should be collected. Further, it is quite difficult to study the effects of malarial infections on the changes of body condition in the field. First, it is hard to detect the exact time of infection and to monitor the acute phase (LaPointe et al., 2012). On the other hand, it is also difficult to find the good measure of body condition that is affected by the parasites. For instance, a study conducted by Hahn et al. (2018) in Bulgaria found that *Plasmodium* P-GRW04 infection (but with low level of parasitaemia) had no effect on the maximum metabolic rate, oxygen consumption, blood haemoglobin concentration or exercise endurance time in the long-distance migrant great reed warblers. Similarly, in other migratory species no difference was found in some of the indicators of immune functions between infected and non-infected individuals, however, local movements of birds on the stopover site (which were associated with these immune function parameters), increased with rising number of parasite species in the host, causing delayed departure from the refuelling site and as a result also a delay in migration (Hegemann et al., 2018). On the other hand, in a Neotropical passerine bird, the rufous-collared sparrow (*Zonotrichia capensis*) the infection with haemosporidians caused increased oxidative stress and weaker antioxidant responses, especially during the breeding season, but had no effect on the body condition (Poblete et al., 2024). Nevertheless, the comparison of the results is difficult, because in this latter mentioned study the parasite species were not determined and their effects were not separated in the models, and most probably the parasite species and lineages differed between the Neotropical and Palearctic breeding and wintering sites.

Finally, in our analysis we could only use the overall prevalence of malaria parasites when we compared infected and non-infected groups, because of the low sample size on the genus, morphospecies and lineage levels. Without the separation of these taxonomic groups, the risk of missing key differences is increasing. Different lineages may have different infectivity and pathogenicity on their avian hosts (Dimitrov et al., 2015), leading also to various effects on the host life and behaviour. For example, in our earlier work on European robins (*Erithacus rubecula*) we also found that individuals infected with haemosporodian parasites arrived later at the stop over site during autumn migration, but the effects of these parasites on fat deposition were lineage-specific (Ágh et al., 2022). So, our results must be interpreted with cautions and further investigations are needed.

## 5 Conclusion

Taken together, in this study we found that three closely related *Acrocephalus* species were infected with altogether 31 different blood parasite lineages and the species composition was age-related. Interestingly, the more virulent *Plasmodium* species exhibited higher prevalence in juvenile birds than adults. Additionally, in two of the three bird species (reed and sedge warblers), the prevalence increased towards the end of migration in juveniles, however we found no relevant differences between the actual body condition of infected and non-infected individuals. In the future, more field sampling is recommended in the breeding season from chicks, juveniles and adults and also from the vectors, to explore the possibility of infection in all age groups and describe the parasite species compositions at the breeding sites. This information could be crucial, as climate change has the potential to alter the geographical distribution of the parasites, potentially disrupting the complex interactions between hosts, parasites and vectors.

## CRediT authorship contribution statement

**Nóra Ágh:** Writing – review & editing, Writing – original draft, Visualization, Methodology, Formal analysis, Data curation, Conceptualization. **Szilvia Pásztory-Kovács:** Writing – review & editing, Writing – original draft, Visualization, Data curation. **Viola Prohászka:** Methodology, Data curation. **Tibor Csörgő:** Writing – review & editing, Resources, Investigation, Data curation. **Eszter Szöllősi:** Writing – review & editing, Writing – original draft, Supervision, Resources, Methodology, Investigation, Conceptualization.

## Ethical standards

All international, national, and institutional guidelines for the care and use of animals were followed. Research was permitted by the Middle-Danube-Valley Inspectorate for Environmental Protection, Nature Conservation and Water Management (under Registration Number KTF: 27251–1/2014). Blood sampling was performed by SPK. (Certificate Registration Number 14/2009, issued by the Institutional Animal Welfare Committee of the University of Veterinary Medicine Budapest).

## Declaration of competing interest

The authors declare that they have no known competing financial interests or personal relationships that could have appeared to influence the work reported in this paper.

## Figures and Tables

**figs1:**
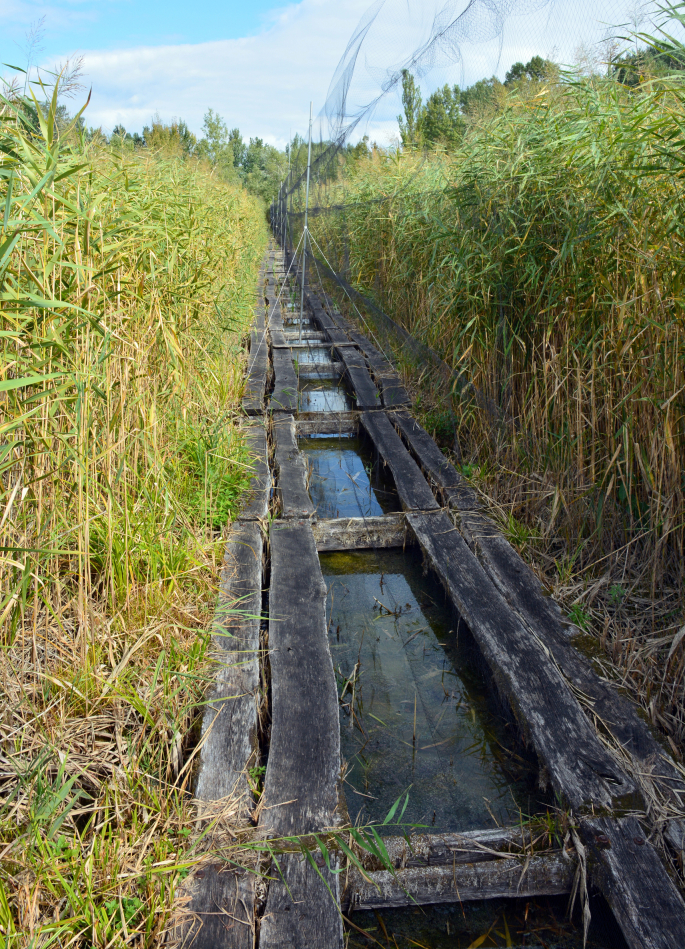
Main habitats of the study site where *Acrocephalus* species were captured during the autumn migration. The ringing station is located on the edge of a protected wetland which is the only natural type habitat patch in the vicinity (Csörgő et al., 2016). (1) Shallow standing water covered by reed, with some willow bushes spreading.

**figs2:**
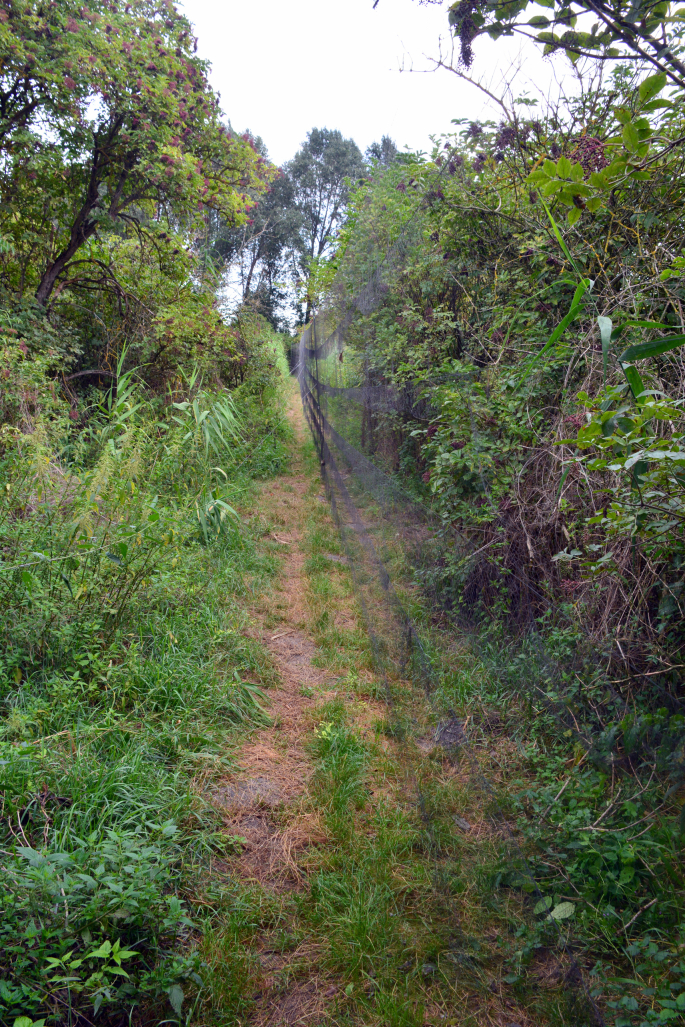
Main habitats of the study site where *Acrocephalus* species were captured during the autumn migration. The ringing station is located on the edge of a protected wetland which is the only natural type habitat patch in the vicinity (Csörgő et al., 2016). (2) Dry reedbed scattered with European elderberry and a few poplar bushes or trees.

**figs3:**
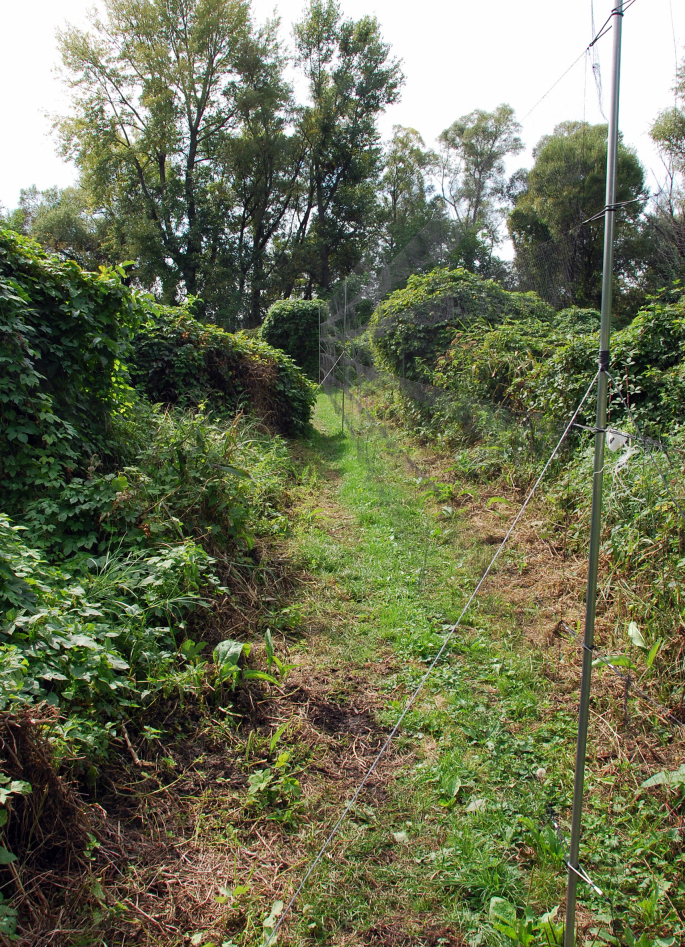
Main habitats of the study site where *Acrocephalus* species were captured during the autumn migration. The ringing station is located on the edge of a protected wetland which is the only natural type habitat patch in the vicinity (Csörgő et al., 2016). (3) dry reedbed with a few elderberries and walnut (*Juglans regia*), partly covered by common hop and more recently also by European blackberry.

**figs4:**
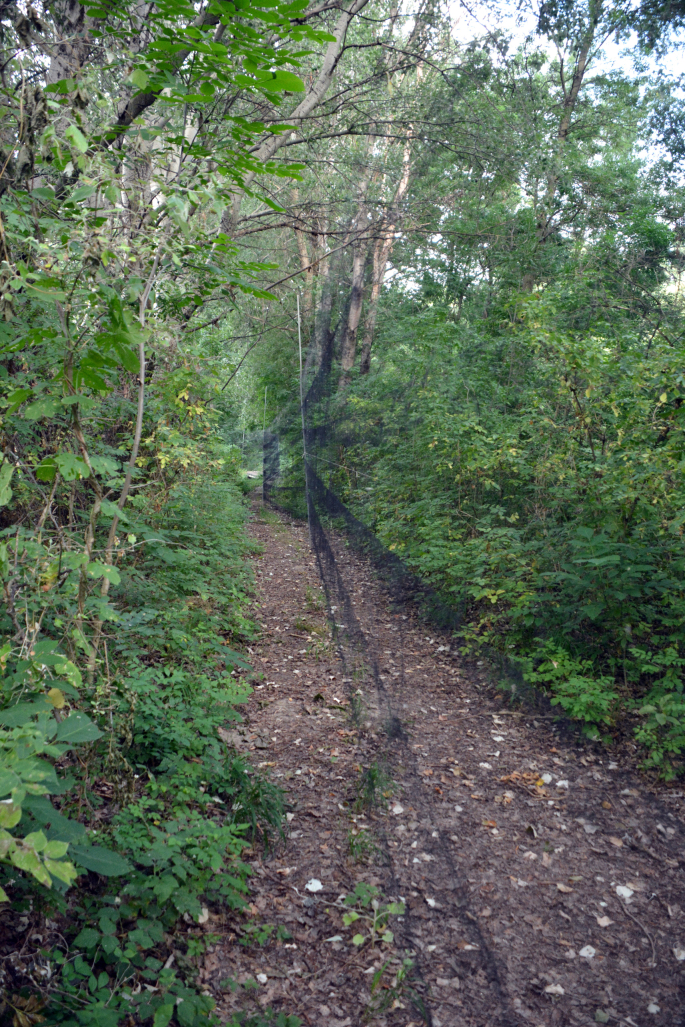
Main habitats of the study site where *Acrocephalus* species were captured during the autumn migration. The ringing station is located on the edge of a protected wetland which is the only natural type habitat patch in the vicinity (Csörgő et al., 2016). (4) A narrow line of poplar trees and willow bushes, surrounded by aquatic habitat mainly reedbed.
