# Supplementary material for: Different age, different blood parasites - Acrocephalus species and their haemosporidian parasites during autumn migration in Central Europe
Source: Int J Parasitol Parasites Wildl. 2025 Jun 1;27:101085. doi: 10.1016/j.ijppaw.2025.101085 (PMC12169794; doi:10.1016/j.ijppaw.2025.101085)
Supplement: Multimedia component 1 [file mmc1.docx]

|  |  |  |  |  | Age: Sex | | | |
| --- | --- | --- | --- | --- | --- | --- | --- | --- |
| Host species | Parasite morphospecies | Host specificity | Lineage (Acc. No.) | Transmission area | ad. ♂ | ad. ♀ | juv.♂ | juv. ♀ |
| Reed Warbler (*Acrocephalus scirpaceus*) | *Haemoproteus balmorali* | specialist (Muscicapidae only) | H-Coll3 (DQ067581) | Europe, Africa, Asia | 1 | 0 | 0 | 0 |
|  | *Haemoproteus pallidus* | generalist | H-Coll2 (DQ060766) | Worldwide | 0 | 0 | **1** | 0 |
|  | *Haemoproteus payevskyi* | generalist | H-RW1 (AF254968) | Europe, Africa, Asia | **2** | **6** | 0 | 0 |
|  | *Haemoproteus belopolskyi* | generalist | H-ARW1 (AF495547) | **Europe** | **4** | **4** | **0** | 0 |
|  |  | generalist | H-MW1 (AF254969) | **Europe**, Africa, Asia | **3** | **2** | **0** | 0 |
|  |  | specialist (*Acrocephalus* spp. only) | **H-RW3 (DQ368364)** | Europe, Asia | **1** | **0** | **0** | 0 |
|  |  | generalist | H-SW1 (AF254967) | Europe, Africa, Asia | **2** | **0** | **0** | 1 |
|  |  | specialist (*Acrocephalus* spp. only) | **H-SW3 (AF495573)** | Europe, Africa, Asia | **1** | **0** | **0** | 0 |
|  | *Haemoproteus spp.* | specialist (Sylviidae only) | H-RW2 (AF495570) | Europe, Africa | 0 | **1** | **1** | 0 |
|  | *Plasmodium circumflexum* | generalist | P-SW5 (AF495574) | Worldwide | **0** | 1 | **1** | **1** |
|  | *Plasmodium elongatum* | generalist | P-GRW06 (DQ368381) | Worldwide | 1 | **0** | 0 | 0 |
|  | *Plasmodium homonucleophilum* | generalist | P-SW2 (AF495572) | Europe, Africa, Asia | **1** | **1** | 1 | **0** |
|  | *Plasmodium  relictum* | generalist | P-GRW04 (AF254975;AY172850) | Worldwide | 0 | **1** | **11** | **9** |
|  |  | generalist | P-GRW11 (AY831748) | Europe, Africa, Asia | 0 | **0** | **1** | **1** |
|  |  | generalist | P-SGS1 (AF495571) | Worldwide | 0 | **0** | **8** | **10** |
|  | *Plasmodium vaughani* | generalist | P-Syat05 (DQ847271) | Worldwide | 0 | 0 | **0** | 1 |
| Marsh Warbler (*Acrocephalus palustris*) | *Haemoproteus pallidus* | generalist | H-Coll2 (DQ060766) | Worldwide | 0 | 0 | **0** | 1 |
|  | *Haemoproteus payevskyi* | generalist | H-RW1 (AF254968) | Europe, Africa, Asia | 2 | **0** | 0 | 0 |
|  | *Haemoproteus belopolskyi* | generalist | H-ARW1 (AF495547) | **Europe** | **1** | **3** | 0 | **0** |
|  |  | generalist | H-MW1 (AF254969) | Europe, Africa, Asia | **0** | 2 | 1 | **0** |
|  |  | generalist | H-SW1 (AF254967) | Europe, Africa, Asia | **2** | 1 | 0 | **0** |
|  | *Plasmodium elongatum* | generalist | P-GRW06 (DQ368381) | Worldwide | **0** | 1 | 1 | **0** |
|  | *Plasmodium  relictum* | generalist | P-GRW11 (AY831748) | Europe, Africa, Asia | 0 | **0** | **4** | **0** |
|  |  | generalist | P-SGS1 (AF495571) | **Worldwide** | **1** | 0 | 7 | **5** |
|  | *Plasmodium spp.* | generalist | P-Delurb4 (EU154346) | Europe, Asia | 0 | 0 | 0 | **1** |
|  |  | generalist | P-RTSR1 (AF495568) | Europe, Africa, Asia | 0 | **1** | 0 | 0 |
|  |  | generalist | P-Sybor10 (DQ368390) | Europe, Africa, Asia | 0 | **1** | 0 | 0 |
|  |  | generalist | P-Coll1 (AY831747) | Europe, Africa | 0 | 0 | **1** | **1** |
|  |  | specialist (*Tchagra senegalus*) | P-TCHSEN01 (MG018672) | Africa | **1** | 0 | 0 | 0 |
| Sedge Warbler (*Acrocephalus schoenobaneus*) | *Haemoproteus majoris* | generalist | H-Parus1 (AF254977) | Europe, Africa, Asia | 0 | 0 | **0** | 1 |
|  | *Haemoproteus payevskyi* | generalist | H-RW1 (AF254968) | Europe, Africa, Asia | **2** | **2** | 1 | **0** |
|  | *Haemoproteus belopolskyi* | generalist | H-ARW1 (AF495547) | **Europe** | 0 | 1 | 0 | 0 |
|  |  | generalist | H-MW1 (AF254969) | Europe, Africa, Asia | 0 | **1** | 0 | 0 |
|  |  | generalist | H-SW1 (AF254967) | Europe, Africa, Asia | 11 | **6** | 0 | 1 |
|  |  | specialist (*Acrocephalus* spp. only) | **H-SW3 (AF495573)** | Europe, Africa, Asia | 3 | **7** | 0 | 0 |
|  | *Plasmodium  ashfordi* | generalist | P-GRW02 (AF254962) | Europe, Africa, Asia | 1 | **0** | 0 | 0 |
|  | *Plasmodium circumflexum* | generalist | P-SW5 (AF495574) | Worldwide | **1** | **1** | **2** | **2** |
|  |  | generalist | P-Turdus1 (AF495576) | **Europe, Africa, Asia** | **0** | **0** | **4** | **4** |
|  | *Plasmodium homonucleophilum* | generalist | P-SW2 (AF495572) | Europe, Africa, Asia | **2** | **1** | **3** | **7** |
|  | *Plasmodium  relictum* | generalist | P-GRW04 (AF254975;AY172850) | Worldwide | **7** | **2** | **7** | **6** |
|  |  | generalist | P-GRW11 (AY831748) | Europe, Africa, Asia | 0 | **0** | 1 | 2 |
|  |  | generalist | P-SGS1 (AF495571) | Worldwide | 0 | **1** | 5 | 3 |
|  | *Plasmodium spp.* | generalist | P-Sybor21 (EF032871) | Europe, Africa | 0 | **1** | 0 | 0 |
|  |  | generalist | P-GRW09 (DQ060773) | Worldwide | **1** | 0 | 0 | 0 |
|  |  | generalist | P-Coll1 (AY831747) | Europe, Africa | **1** | 0 | 0 | 0 |
|  |  | generalist | P-BT7 (AY393793) | Worldwide (exp. Africa) | 0 | 0 | 0 | **2** |
|  |  | generalist | P-Sybor02 (DQ368392) | Europe, Africa, Asia | 0 | 0 | 0 | **1** |
|  |  | new | P-SW6 (PV491362) | Europe | 0 | 0 | 0 | **1** |

Table S2: Prevalence of the two haemosporidian genera by age and sex groups separately in the studied three *Acrocephalus* species. The prevalence and the 95% confidence intervals were estimated with the Quantitative Parasitology 3.0 program.
*The adult total category means the samples from 2012 and 2013 together. In the case of juveniles, we did not calculate the prevalence of Haemoproteus infection in sex groups separately due to the low sample sizes.*

| Genus | Age/sex | Non-infected | Infected | Prevalence | 95% CI |
| --- | --- | --- | --- | --- | --- |
| Reed Warbler (*Acrocephalus scirpaceus*) | | | | | |
| Total | Adult total | 25 | 33 | 0.569 | 0.439;0.691 |
|  | Adult 2012 | 19 | 27 | 0.587 | 0.434;0.719 |
|  | Juvenile | 144 | 28 | 0.163 | 0.113;0.226 |
|  | Male | 61 | 13 | 0.176 | 0.101;0.283 |
|  | Female | 83 | 15 | 0.153 | 0.091;0.239 |
| Haemoproteus | Adult | 25 | 27 | 0.519 | 0.384;0.655 |
|  | Adult 2012 | 19 | 23 | 0.548 | 0.392;0.692 |
|  | Juvenile | 144 | 3 | 0.020 | 0.006;0.060 |
| Plasmodium | Adult | 25 | 5 | 0.167 | 0.068;0.347 |
|  | Adult 2012 | 19 | 3 | 0.136 | 0.038;0.338 |
|  | Juvenile | 144 | 24 | 0.143 | 0.097;0.205 |
|  | Male | 61 | 11 | 0.153 | 0.083;0.256 |
|  | Female | 83 | 13 | 0.135 | 0.076;0.218 |
| Marsh Warbler (*Acrocephalus palustris*) | | | | | |
| Total | Adult | 51 | 21 | 0.292 | 0.193;0.409 |
|  | Adult 2012 | 46 | 13 | 0.220 | 0.130;0.346 |
|  | Juvenile | 109 | 34 | 0.238 | 0.174;0.314 |
|  | Male | 50 | 21 | 0.296 | 0.196;0.415 |
|  | Female | 59 | 13 | 0.181 | 0.104;0.290 |
| Haemoproteus | Adult | 51 | 11 | 0.177 | 0.098;0.297 |
|  | Adult 2012 | 46 | 8 | 0.148 | 0.070;0.267 |
|  | Juvenile | 109 | 2 | 0.018 | 0.003;0.066 |
| Plasmodium | Adult | 51 | 5 | 0.089 | 0.036;0.194 |
|  | Adult 2012 | 46 | 2 | 0.042 | 0.007;0.142 |
|  | Juvenile | 109 | 20 | 0.156 | 0.083;0.264 |
|  | Male | 50 | 13 | 0.206 | 0.121;0.324 |
|  | Female | 59 | 7 | 0.106 | 0.051;0.203 |
| Sedge Warbler (*Acrocephalus schoenobaneus*) | | | | | |
| Total | Adult | 25 | 55 | 0.688 | 0.575;0.783 |
|  | Adult 2012 | 16 | 37 | 0.698 | 0.557;0.813 |
|  | Juvenile | 143 | 43 | 0.231 | 0.174;0.298 |
|  | Male | 70 | 16 | 0.186 | 0.115;0.284 |
|  | Female | 73 | 27 | 0.270 | 0.189;0.364 |
| Haemoproteus | Adult | 25 | 33 | 0.569 | 0.439;0.691 |
|  | Adult 2012 | 16 | 23 | 0.590 | 0.422;0.733 |
|  | Juvenile | 143 | 3 | 0.021 | 0.006;0.060 |
| Plasmodium | Adult | 25 | 18 | 0.419 | 0.277;0.570 |
|  | Adult 2012 | 16 | 11 | 0.407 | 0.237;0.598 |
|  | Juvenile | 143 | 39 | 0.214 | 0.159;0.280 |
|  | Male | 70 | 15 | 0.176 | 0.106;0.275 |
|  | Female | 73 | 24 | 0.247 | 0.169;0.345 |

Table S3: Estimated prevalence in relation to year, sampling period and sex in juvenile age groups of each species separately. Effects (shown as χ^2^) are presented as analysis of deviance tables with type‐2 sums of squares for the generalized linear models.

| Species | Predictor | χ^2^ | p-value |
| --- | --- | --- | --- |
| Reed Warbler (*Acrocephalus scirpaceus*) | Year (factor) | **6.355** | **0.012** |
|  | Sampling period (factor) | 6.196 | 0.288 |
|  | Sex | 0.020 | 0.888 |
| Marsh Warbler (*Acrocephalus palustris*) | Year (factor) | 0.120 | 0.729 |
|  | Sampling period (factor) | 6.317 | 0.389 |
|  | Sex | 2.994 | 0.083 |
| Sedge Warbler (*Acrocephalus schoenobaneus*) | Year (factor) | 0.494 | 0.482 |
|  | Sampling period (factor) | **17.994** | **0.006** |
|  | Sex | 1.830 | 0.176 |
